# Dietary prevention of antibiotic‐induced dysbiosis and mortality upon aging in mice

**DOI:** 10.1096/fj.202402262R

**Published:** 2024-12-10

**Authors:** Kelsey M. Smith, Sarah G. Francisco, Ying Zhu, Tanya LeRoith, Meredith L. Davis, Jimmy W. Crott, Kathryn Barger, Andrew S. Greenberg, Donald E. Smith, Allen Taylor, Laxmi Yeruva, Sheldon Rowan

**Affiliations:** ^1^ Jean Mayer USDA Human Nutrition Research Center on Aging Tufts University Boston Massachusetts USA; ^2^ The Friedman School of Nutrition Science & Policy Tufts University Boston Massachusetts USA; ^3^ Department of Biomedical Sciences and Pathobiology VA‐MD College of Veterinary Medicine, Virginia Tech Blacksburg Virginia USA; ^4^ Department of Pathology & Laboratory Medicine Boston University School of Medicine Boston Massachusetts USA; ^5^ USDA‐ARS, Microbiome and Metabolism Research Unit Arkansas Children's Nutrition Center Little Rock Arkansas USA

**Keywords:** aging, antibiotic, gastrointestinal disease, glycemic index, inflammation, microbiome, resistant starch, survival

## Abstract

Oral antibiotic use is both widespread and frequent in older adults and has been linked to dysbiosis of the gut microbiota, enteric infection, and chronic diseases. Diet and nutrients, particularly prebiotics, may modify the susceptibility of the gut microbiome to antibiotic‐induced dysbiosis. We fed 12‐month‐old mice a high glycemic (HG) or low glycemic (LG) diet with or without antibiotics (ampicillin and neomycin) for an additional 11 months. The glycemic index was modulated by the ratio of rapidly digested amylopectin starch to slowly digested amylose, a type‐2‐resistant starch. We observed a significant decrease in survival of mice fed a HG diet containing antibiotics (HGAbx) relative to those fed a LG diet containing antibiotics (LGAbx). HGAbx mice died with an enlarged and hemorrhagic cecum, which is associated with colonic hyperplasia and goblet cell depletion. Gut microbiome analysis revealed a pronounced expansion of *Proteobacteria* and a near‐complete loss of *Bacteroidota* and *Firmicutes* commensal bacteria in HGAbx, whereas the LGAbx group maintained a population of *Bacteroides* and more closely resembled the LG microbiome. The predicted functional capacity for bile salt hydrolase activity was lost in HGAbx mice but retained in LGAbx mice. An LG diet containing amylose may therefore be a potential therapeutic to prevent antibiotic‐induced dysbiosis and morbidity.

## Graphical abstract

Aged mice fed high glycemic index diets and treated with oral antibiotics to deplete commensal gut microbiota developed severe gut dysbiosis, characterized by very low alpha diversity and high prevalence of antibiotic‐resistant potentially pathogenic bacteria. These mice died suddenly of gastrointestinal disease. If mice were instead fed low glycemic index diets with the same antibiotics, the mice had attenuated dysbiosis with higher alpha diversity, more commensal gut microbiota, and comparable long‐term survival to nonantibiotic‐treated mice.

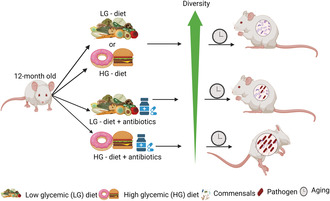

**Abbreviations**
ANOVA: analysis of variance
ASV: amplicon sequence variant
FDR: false discovery rate
H&E: hematoxylin and eosin
HG: high glycemic diet
HGAbx: high glycemic diet with antibiotics
IQR: interquartile range
LG: low glycemic diet
LGAbx: low glycemic diet with antibiotics
MAC: microbial‐accessible carbohydrate
NAD: nicotinamide adenine dinucleotide
PC: principal component
PERMANOVA: permutational multivariate analysis of variance
rDNA: ribosomal DNA
SCFA: short‐chain fatty acid
TCA: tricarboxylic acid cycle

## 1 INTRODUCTION

Promoting health and preventing chronic disease throughout the lifespan is becoming increasingly important as the global population of older adults continues to expand. The gut microbiome of older adults has decreased diversity, altered composition, and increased vulnerability to colonization by pathogens.^1^ This dysbiotic phenotype has been shown to promote systemic inflammation and chronic disease.^2^ The environmental factors that alter the gut microbiome, including diet and medication use, are promising targets for developing strategies to reduce the prevalence and severity of age‐related diseases, and consequently to improve well‐being and longevity of older adults.

Antibiotics are an important potential modifier for the relationship between gut microbiota and health outcomes in older adults. Broad‐spectrum antibiotics have profound effects on gut microbiota composition and function,^3^ leading to increased susceptibility to enteric infection,^4^ increased microbial translocation,^5^ and decreased SCFA production. Health outcomes associated with antibiotic use include diverse conditions such as diarrhea, obesity, inflammatory bowel disease, depression, and age‐related macular degeneration.^6^, ^7^, ^8^, ^9^ Frequent or long‐term antibiotic use, which is prevalent among older adults,^10^ may be a significant contributor to age‐related disease. Individuals prescribed antibiotics for life‐long use are at particularly high risk for the development of antibiotic‐resistant infections and side effects of the antibiotics, both of which can be life‐threatening.^11^, ^12^

Dietary carbohydrates are another important modifier of health and the gut microbiome. Previous research has shown that increased intake of sugar and refined starches and concomitant reduced intake of dietary fiber in older adults is associated with elevated risk of inflammatory disease mortality.^13^ Our work has found that a diet containing mainly rapidly digestible amylopectin, as opposed to slowly digestible amylose, leads to obesity, reduced glucose sensitivity, and retinopathy in older mice.^14^

Additionally, dietary carbohydrates have been found to modify the effect of antibiotics on gut microbiota composition and function. Inulin, pectin, and oligosaccharides alter the effect of antibiotics on microbiota composition and function in in vitro fermentation models.^15^, ^16^ Prebiotic diet composition appears to have a critical role in antibiotic‐induced dysbiosis. In gnotobiotic mice reconstituted with human feces, recovery of bacteria alpha diversity and *Bacteroides* genera after antibiotic treatment was compromised in mice fed a diet lacking microbial‐accessible carbohydrates (MACs).^17^ Conversely, supplementing mice with a MAC‐rich diet containing seven plant fibers rescued antibiotic‐induced dysbiosis and gut redox dysregulation.^18^

The interactions between dietary carbohydrates, antibiotic use, and health outcomes have only been minimally explored in the context of aging. To evaluate these relationships directly, we fed 12‐month‐old mice a high glycemic (HG) or low glycemic (LG) diet with or without antibiotics for an additional 11 months and evaluated gut microbiome composition and function, gut health, and survival. We found that mice fed a HG diet and antibiotics developed severe antibiotic‐induced dysbiosis and died from an acute gastrointestinal inflammatory disease, whereas mice fed LG diets and antibiotics were protected from death and had attenuated dysbiosis.

## 2 MATERIALS AND METHODS

### 2.1 Study design and animal care

**FIGURE 1:**
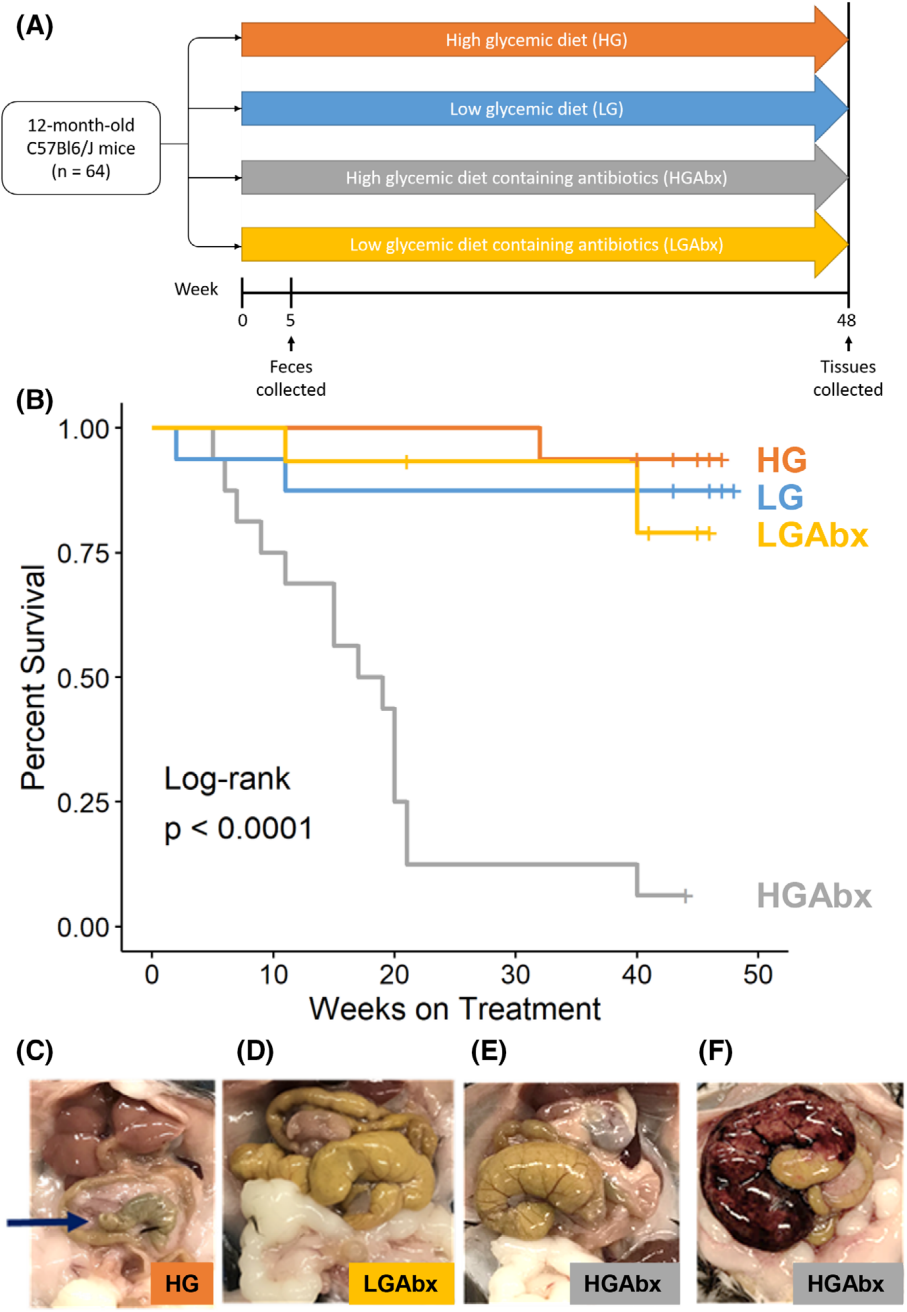
Lethality caused by HGAbx treatment. (A) Schematic of study design. (B) Kaplan–Meier survival plots show the HGAbx treatment significantly decreased survival compared with all other groups. (C–F) Representative photos of ceca taken from different groups of mice show that control HG mice had normal‐sized ceca (C, arrow), while LGAbx mice had enlarged, but otherwise normal ceca (D). Only two HGAbx mice survived past 18 months and had ceca resembling the LGAbx mice (E). All other HGAbx that died had enlarged and hemorrhagic ceca upon necropsy (F). Sample size is *n* = 16 for each group.

Animal work was performed at the Tufts University HNRCA and approved by the Tufts University IACUC in adherence with the National Institutes of Health guide for the care and use of Laboratory animals (NIH Publications No. 8023, revised 1978). Male C57BL/6J retired breeder mice were obtained at 11 months of age (Jackson Laboratories, Bar Harbor, ME, USA) and individually housed in plastic micro‐isolator cages with ad libitum access to water. Mice were acclimated to the facility for 1 month prior to treatment and provided standard chow diet during this time (Teklad 2916 irradiated diet, Envigo, USA). At 12 months of age, mice were randomly assigned to a HG or LG diet with or without antibiotics (*n* = 16/group, 64 total, Figure 1A). All mice were weighed weekly. Body composition analysis was performed at 34 weeks of feeding using an EchoMRI‐700 to measure fat mass and lean body mass. After 11 months of treatment, mice were fasted for 6 h and humanely euthanized via cardiac puncture. Whole blood was collected via cardiac puncture and plasma was stored at −80°C. The details of the study have been previously published in a thesis dissertation by Smith.^19^

### 2.2 Diets and feeding

All diets have been previously published by our group and others.^14^, ^20^ The HG and LG diets are isocaloric and identical apart from the starch composition, which is 100% rapidly digested amylopectin (Amioca starch, Ingredion Inc., Bridgewater, NJ) in the HG diet and 70% slowly digested amylose/30% amylopectin (Hylon VII starch, Ingredion Inc., Bridgewater, NJ) in the LG diet. Diet composition was 542 g/kg starch, 200 g/kg casein, 85 g/kg sucrose, 56 g/kg soybean oil, 50 g/kg wheat bran, 2 g/kg DL‐methionine, 10 g/kg vitamin mix, and 35 g/kg mineral mix. Macronutrient energy percentages were 65% carbohydrate, 21% protein, and 14% fat for both HG and LG diets. Diets containing antibiotics were formulated with 320 mg ampicillin + 640 mg neomycin/kg diet (Goldbio, St. Louis, MO, USA), based on published dosing protocols and taking into account average daily food and water intake.^21^ All diets were formulated by Bio‐Serv (Frenchtown, NJ). Mice were group pair‐fed to ensure equal consumption.

### 2.3 Intraperitoneal glucose tolerance test

Glucose tolerance was measured via intraperitoneal glucose tolerance test after 37 weeks of treatment. Mice were fasted for 6 h in clean cages with access to water. A razor blade was used to make a horizontal cut in the lateral tail vein and fasting blood glucose was measured via OneTouch Ultra2 glucometer (LifeScan, Inc.). Mice then received an intraperitoneal injection of 1 g/kg BW D‐(+)‐glucose (≥99.5%) (Sigma) via a 25–5/8–gauge syringe. Blood glucose was measured via original tail cut at 15, 30, 60, 90, and 120 min postinjection.

### 2.4 Plasma cytokine analysis

Plasma proinflammatory cytokine concentrations were determined using the MSD U‐PLEX Adipokine Combo 1 (Meso Scale Discovery) on the SECTOR Imager 2400.

### 2.5 Microbiome collection and analysis

After 5 weeks of treatment, fecal pellets were collected from empty sterile cages and stored at −80°C prior to gut microbiota sequencing. Samples were randomly selected from each group, with a sample size of five each for control groups and 10 each for Abx groups. Microbial DNA was isolated using the QiaAMP PowerFecalPro DNA kit (QIAGEN, Hilden, Germany). 16S rDNA libraries were prepared using the Earth Microbiome Project protocol with the V4‐V5 primer region as described previously.^22^ Demultiplexed paired‐end reads were imported into QIIME2 (v2021.8) for quality control, feature table construction, and computation and significance testing of alpha‐ and beta‐diversity.^23^, ^24^, ^25^ The DADA2 pipeline was used for quality filtering and table construction.^26^ As the quality scores were consistently high across the length of the sequence, sequences were not trimmed. Prior to diversity analyses, samples were rarefied to the minimum read count across all samples, which was ~34 000 in our sample set. Taxonomic assignment was performed by training a Naïve Bayes Classifier^27^ on the SILVA_138 reference database specific to the V4‐V5 region.^28^, ^29^, ^30^

### 2.6 Bioinformatics

Differential abundance of taxa, pathways, and genes was determined using the MaAsLin2 package (v1.4.0) in R.^31^ Briefly, a matrix of annotated and unfiltered raw count data was imported into R and evaluated using the default MaAsLin2 settings, including filtering out taxa/pathways/genes with a sample prevalence of less than 10%, total‐sum‐scaling normalization, log transformation, and the linear model analysis method. Two‐way analysis was performed using diet, antibiotic treatment, and an interaction term as fixed effects. Taxa with significant interaction terms were further evaluated for pairwise comparisons with HG as the reference group. Features were considered significantly different if the false‐discovery rate corrected *q*‐value was less than 0.25.

Microbiome function was predicted using PICRUSt2 software (v2.4.1) using the default settings.^32^ Due to the high abundance of rare taxa in the Abx groups, sequence data were not prevalence‐ or abundance‐filtered prior to analysis.

### 2.7 Statistical analysis

Group comparisons of continuous variables were performed by first checking the assumptions for parametric tests followed by the appropriate analysis. Assumptions were tested using the Shapiro–Wi lk normality test and Levene's test for homogeneity of variance from the rstatix package (v0.7.0) in R. Parametric data were analyzed via two‐way ANOVA when HGAbx data are available, and one‐way ANOVA when data are only available for HG, LG, and LGAbx. Nonparametric two‐way analysis was performed using a rank‐based ANOVA robust to non‐normal data (R, WRS2::t2way, v1.1‐3). Nonparametric univariate data were analyzed via Kruskal–Wallis followed by Dunn's test with Holm's correction when significant. Survival was evaluated using the survival package (v3.2‐10) in R using the log‐rank hypothesis test. Analyses were performed using R version 4.0.5.

### 2.8 Data visualization

Boxplots were prepared using the ggpubr package (v0.4.0), and boxes indicate median and interquartile range (IQR). Boxplot whiskers extend to the minimum and maximum data points within 1.5*(IQR). Some boxplots were generated using BoxPlotR.^33^ Superplots were generated using SuperPlotsOfData.^34^ Heatmaps were prepared using the ComplexHeatmap package (v2.6.2). The heatmap of differential abundance of taxa shows all significantly differentially abundant taxa with an abundance of at least 1% in at least one sample. The heatmap of pathways shows the 50 most differentially abundant taxa based on lowest FDR‐corrected p‐value (defined as the q‐value) across all treatment groups for that pathway. The relative abundance shown in both heatmaps was calculated by dividing the count of a feature by the sum of counts for the sample. Beta‐diversity of taxa was visualized using Emperor software built into QIIME2. Beta‐diversity of pathways was visualized using the vegan package (v2.5‐7) in R.

### 2.9 Colon histology and mRNA analysis

At necropsy, colons were removed and flushed with ice‐cold PBS containing protease and phosphate inhibitors (Roche, Indianapolis, IN). A 1–2 cm segment was collected from the distal colon and fixed in 10% formalin. Paraffin sections were collected and stained with hematoxylin, eosin, and alcian‐blue. Representative images of sections were photographed at 20× or 60× magnification on an Olympus BX51 photomicroscope equipped with a DP70 digital camera with 24‐bit depth. Images were scored blindly by a pathologist (Dr. Tanya LeRoith, veterinary pathologist). Gland lengths and goblet cell numbers were averaged over 6–10 glands.

Colonic mucosa was gently scraped from the apical surface of the colon using a glass slide and flash‐frozen in liquid nitrogen prior to storage at −80°C. Total RNA was isolated from colonic mucosa using TRIzol® reagent (Thermo Fisher) and cDNA was synthesized using the Superscript® IV First‐Strand Synthesis System (Thermo Fisher). Target transcripts were amplified using Power up SYBR® Green Master Mix and an Applied Biosystems Plus real‐time PCR system (Thermo Fisher). The expression of each target gene was normalized against Gapdh and relative expression compared with the LG group was calculated.

Primer sequences are as follows:

Ocln_FWD 5′‐GTGAATGGGTCACCGAGGG; Ocln_RVS 5′‐AGATAAGCGAACCTGCCGAG
Tjp1_FWD 5′‐GCCGCTAAGAGCACAGCAA; Tjp1_RVS 5′‐GCCCTCCTTTTAACACATCAGA
Tjp2_FWD 5′‐AGCTTGTAGTTCTGAGCCGC; Tjp2_REV 5′‐GCTCCCATATCACCTCCTCC
Cldn1_FWD 5′‐GGGGACAACATCGTGACCG; Cldn1_RVS 5′‐AGGAGTCGAAGACTTTGCACT
Cldn2_FWD 5′‐ATGCCTTCTTGAGCCTGCTT; Cldn2_REV 5′‐CAGTGTCTCTGGCAAGCTGA
TNF_FWD 5′‐AGGGTCTGGGCCATAGAACT; TNF_RVS 5′‐CCACCACGCTCTTCTGTCTAC
IL‐1β_FWD 5′‐TGAAGCAGCTATGGCAACTG; IL‐1β_RVS 5′‐AGGTCAAAGGTTTGGAAGCA
IL‐10_FWD 5′‐GCTCTTACTGACTGGCATGAG; IL‐10_RVS 5′‐CGCAGCTCTAGGAGCATGTG
Gapdh_FWD 5′‐TTGATGGCAACAATCTCCAC; Gapdh_RVS 5′‐CGTCCCGTAGACAAAATGGT

## 3 RESULTS

### 3.1 Mice fed a HG diet containing antibiotics died with GI disease

Mice aged 12 months were fed either a LG or HG diet with or without antibiotics (Abx, ampicillin, and neomycin) for 48 weeks (Figure 1A). Diets were identical apart from starch composition, which was 100% rapidly digested amylopectin in the HG diet and 30% amylopectin/70% slowly digested amylose in the LG diet.

Mice in the HGAbx group suffered unexpected premature death—of the 16 mice in the group, 14 died within the first 5 months of treatment (Figure 1B). Affected mice died within 2 days of first showing symptoms (decreased movement, food intake, and responsiveness as well as abdominal swelling), and upon necropsy we observed an enlarged and hemorrhagic cecum (Figure 1F). Enlarged ceca have been previously observed in antibiotic‐treated mice,^35^ germ‐free mice,^36^ and mice fed high amounts of resistant starch,^37^ but not in a pathological context. We observed cecal enlargement in LGAbx mice (Figure 1D) and unaffected HGAbx mice (Figure 1E), but the most of the LGAbx mice did not experience hemorrhage and death. Other organ systems appeared normal during necropsy.

We monitored body weight and adiposity over the course of aging to determine whether antibiotic treatment modified body composition in a way that could impact mortality. As we have previously reported, mice fed HG diets gained weight and became obese during aging, while mice fed LG diets maintained body weight during aging (Figure S1A,B). Antibiotic treatment modified weight gain and body composition. LGAbx mice had increased body weight relative to LG mice which was contributed by both increased lean and fat mass (Figure S1A,B). HGAbx mice had decreased body weight relative to HG mice, which was accounted for by decreased fat mass (Figure S1A,B). Food intake was not different, as mice were pair‐fed. However, these results were not statistically significant due to the premature death of all but two mice at 36 weeks on study. Both LG and LGAbx mice showed significantly improved insulin sensitivity and glucose tolerance relative to HG mice (Figure S2A,B).

### 3.2 Antibiotic‐induced gut dysbiosis is attenuated by LG diet

**FIGURE 2:**
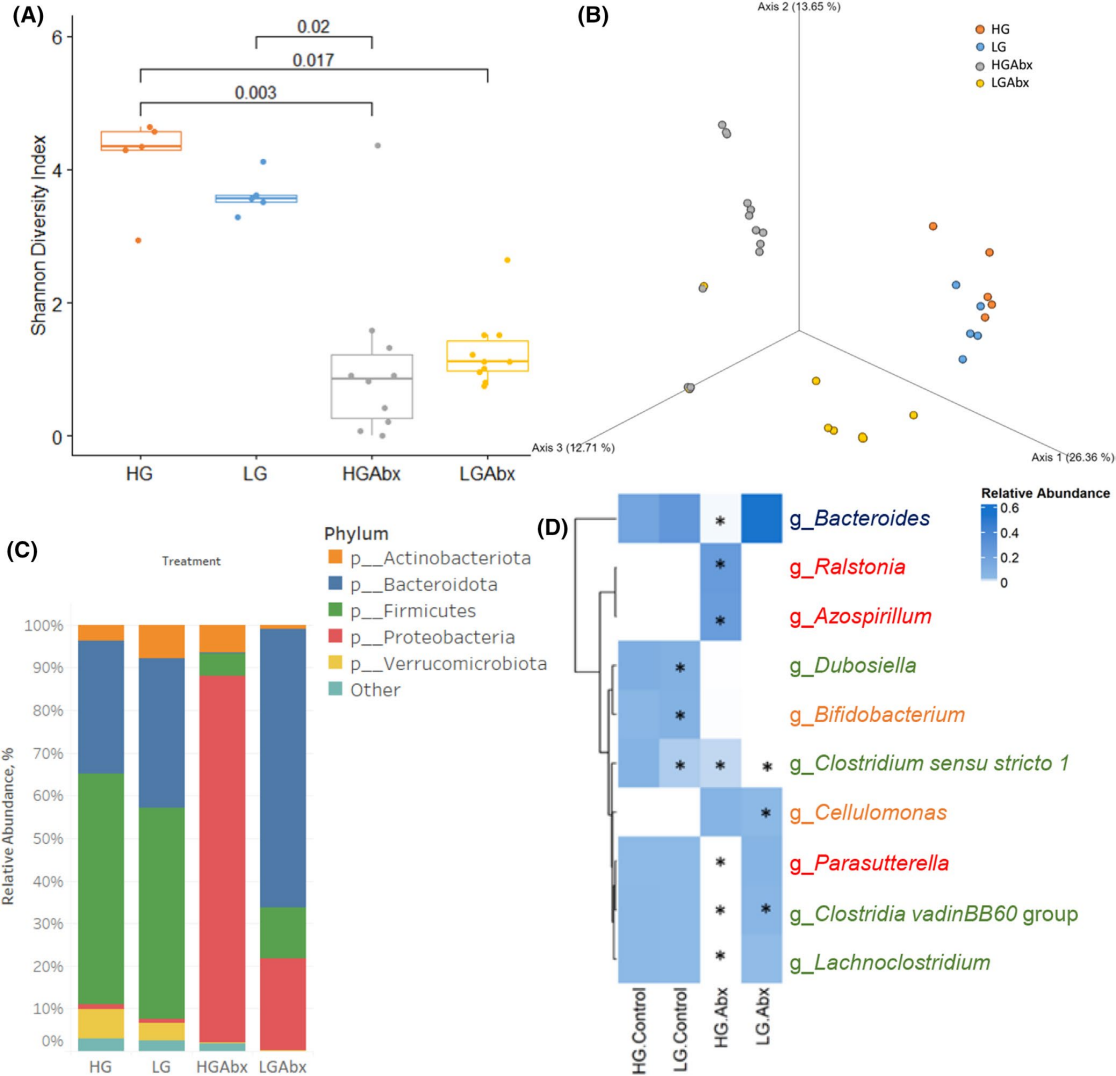
Antibiotic‐induced dysbiosis in aged mice. (A) Antibiotic treatment reduced alpha diversity. Values denote p‐values for pairwise comparisons, as assessed by Kruskal–Wallis followed by Dunn's test with Holm's correction. (B) Microbial composition differed significantly (*p* < .001 by PERMANOVA) across all treatment groups, as determined via Bray–Curtis beta‐diversity analysis. (C) Antibiotic treatment promoted a relative expansion of Proteobacteria phyla and loss of commensal bacterial phyla in HGAbx mice, while LGAbx mice maintained Bacteroidota. (D) Heatmap of average relative abundance of genera (minimum abundance of 1% in at least one group) with a significant antibiotic‐diet interaction. Asterisk denotes significantly differential abundance (adjusted *p*‐value <.25). Genera are colored according to their phyla as in (C). Sample size: *n* = 5, LG, HG; *n* = 10 LGAbx, HGAbx.

In order to assess the impact of antibiotic treatment and diet on gut microbiome composition, we collected fecal samples after 5 weeks of feeding the treatment diet and performed 16S rDNA microbiome sequencing. We found that alpha‐diversity was significantly decreased in both antibiotic‐treated groups (Figure 2A). Beta‐diversity analysis indicated that microbiome composition differed significantly across all treatment groups (Figure 2B, PERMANOVA *p* < .001). HG and LG control microbiomes most closely resembled each other, while the distance between HG and HGAbx was larger than the distance between LG and LGAbx groups.

At the phylum level, both antibiotic‐treated groups had a greater relative abundance of Proteobacteria and less Firmicutes (Figure 2C). HGAbx mice experienced a complete absence of Bacteroidota, but LGAbx mice were able to maintain a population of Bacteroidota. A two‐way analysis of differential abundance of taxa was performed with Abx and diet as main effects. Abx treatment led to a significant decrease in known commensal bacteria including *Muribaculaceae*, *Akkermansia*, and *Lactobacillus* (Figure S3). Aerobic proteobacteria including *Ralstonia* and *Azospirillum* were promoted by the Abx treatment (Figure S3). Pairwise comparisons were made in taxa with significant interactions of Abx and diet (Figure 2D). LG diet significantly modified the effect of antibiotics on abundance of multiple taxa. Commensal bacteria across multiple taxa such as *Bacteroides*, *Parasutterella*, *Clostridia vadinBB60* group, and *Lachnoclostridia* were all present in LGAbx at levels that were as high as or higher than control groups and were not present in HGAbx mice. Conversely, potentially pathogenic Proteobacteria such as *Ralstonia* and *Azospirillum*, were present only in HGAbx mice but not LGAbx mice.

A cardinal feature of a dysbiotic microbiome is the overabundance of a single kind of bacteria that is normally present at very low amounts or absent from a normal microbiome. Evaluation of taxa in individual HGAbx or LGAbx mice showed that all of the HGAbx microbiomes were dominated by one or two taxa (Figure S4). In some HGAbx mice, greater than 95% of all taxa consisted of a single species. We identified several potential pathogens in HGAbx mice such as *Ralstonia*, *Enterobacteriaceae*, *Azospirillum*, *Acinetobacter*, *Cellulomonas*, and *Cupriavidus*. LGAbx, while still having higher levels of Proteobacteria than control mice, were not dominated by monocultures of bacteria (Figure S4).

### 3.3 Rescue of antibiotic‐induced dysbiotic microbial function by LG diet

**FIGURE 3:**
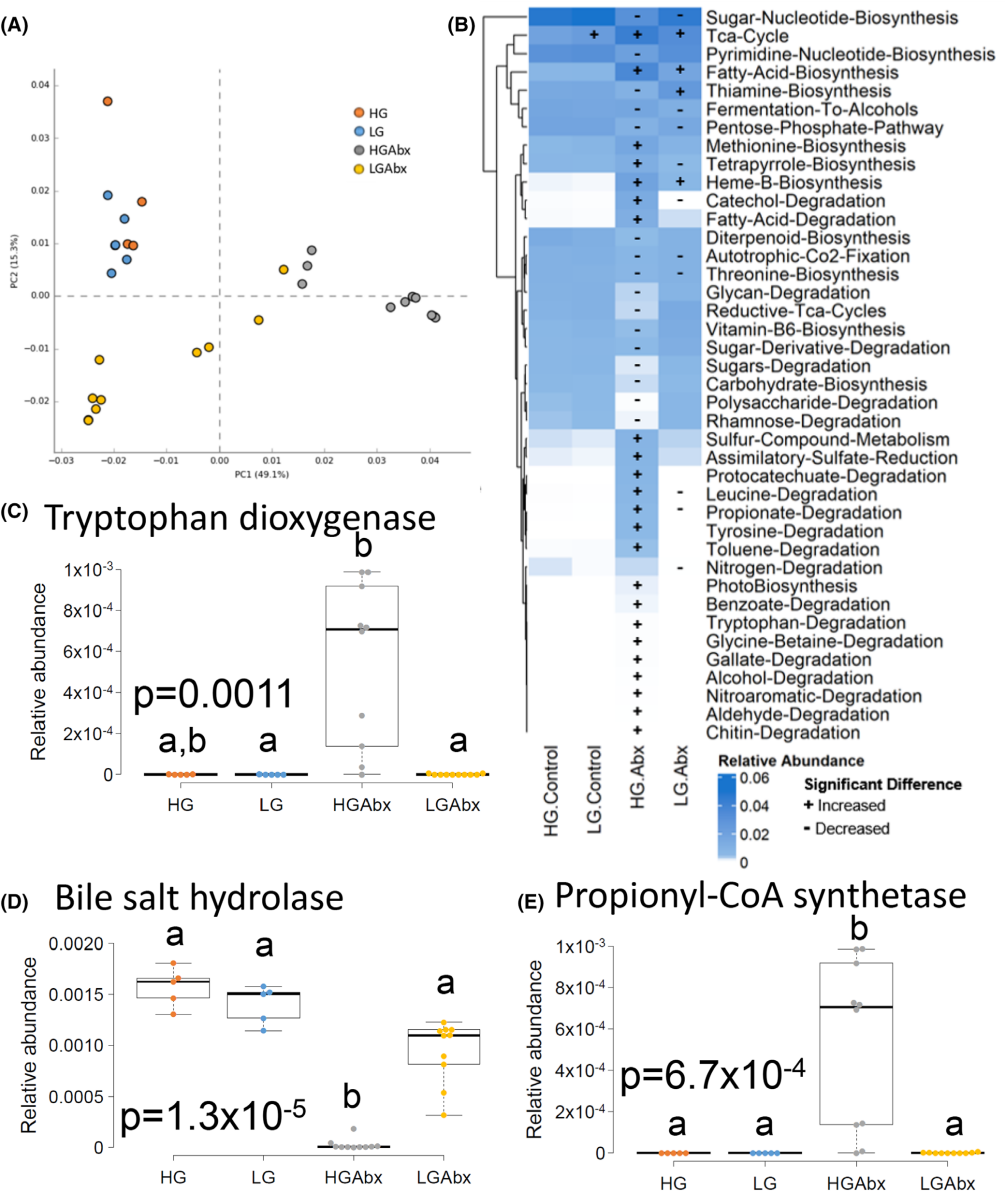
Rescue of antibiotic‐induced dysbiotic microbial function by LG diet. (A, B) Microbiota pathways were inferred using PICRUSt2 and compared across groups, as shown via principal component analysis (A) or via heatmaps of the top 40 differentially abundant pathways (B). Heatmaps are colored by relative abundance of pathway and significant differences are indicated by arithmetic sign. (C–E) Boxplots of genes encoding tryptophan dioxygenase (C), bile salt hydrolase (D), or propionyl‐CoA‐synthetase (E). *p*‐values are indicated for Kruskal–Wallis analysis with different letters indicating *p* < .05 from post hoc analysis using Dunn's test with Holm's correction. Sample size *n* = 5, LG, HG; *n* = 10 LGAbx, HGAbx.

Gut microbiome function was predicted from 16S rRNA sequencing using the PICRUSt2 (Phylogenetic Investigation of Communities by Reconstruction of Unobserved States) software. Despite significantly different gut microbiome compositions, control HG and LG groups showed functional redundancy of their predicted metagenomes, while HGAbx predicted metagenomes were most divergent from control groups (Figure 3A). LGAbx predicted metagenomes were more similar to LG‐predicated metagenomes, particularly along the PC1 axis (Figure 3A). Visualization of differentially abundant pathways using heatmaps showed that 39 of the top 40 differential pathways were significantly altered by HGAbx treatment (Figure 3B). Of these 39 pathways, 31 were not significantly different in LGAbx groups, indicating an attenuation of dysbiotic pathways.

We further explored three specific pathways and enzyme abundances of special interest to gut and systemic health. First, we evaluated tryptophan degradation, which was increased in the HGAbx group (Figure 3B). Tryptophan can be degraded or metabolized along different pathways to form indoles and kynurenines.^38^ We found that the rate‐limiting enzymes for degradation of tryptophan to kynurenine, tryptophan dioxygenase (EC: 1.13.11.11), and arylformamidase (EC: 3.5.1.9) were significantly upregulated in the HGAbx group, while the rate‐limiting enzyme for the degradation of tryptophan to indole, tryptophanase (EC: 4.1.99.1), was lowest in the HGAbx group (Figures 3C and S5). We also found that kynureninase (EC: 3.7.1.3), which further degrades kynurenine to anthranilate was significantly upregulated in the HGAbx group (Figure S5C). Anthranilate can act as a signaling factor, modulating the pathogenicity of bacteria.^39^

Next, we evaluated levels of bile salt hydrolase (EC: 3.5.1.24), which deconjugates primary bile acids from glycine and taurine—an obligatory step in the formation of secondary bile acids. Bile salt hydrolase was absent from most mice in the HGAbx group and present in almost control levels in the LGAbx group (Figure 3D). Secondary bile acids have important effects throughout the body, including modulation of host immune and metabolic health and prevention of enteric infection.^40^

Finally, we evaluated genes involved in metabolism of short‐chain fatty acids, products of microbial carbohydrate fermentation that are known to have many beneficial effects throughout the host including improved glycemic control, increased satiety signaling, anti‐obesogenic effects, and decreased gut inflammation and permeability. The HGAbx group had significantly increased capacity for propionate degradation, which was completely reversed in the LGAbx group (Figure 3B) and was reflected by HGAbx‐specific expression of propionyl‐CoA synthetase (EC: 6.2.1.1) (Figure 3E). Fermentative pathways leading to acetate, butyrate, or propionate were not significantly altered in the HGAbx group.

### 3.4 Colonic hyperplasia and goblet cell loss in HGAbx‐associated mortality

**FIGURE 4:**
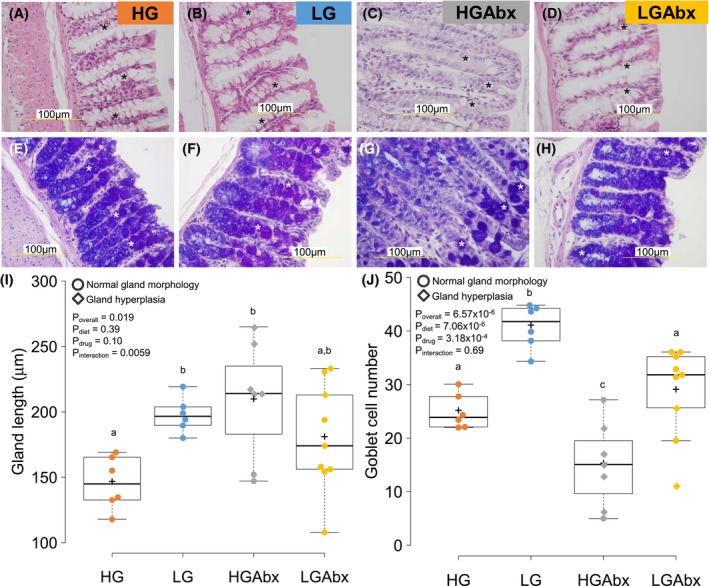
Colonic hyperplasia and loss of goblet cells in HGAbx‐treated mice. (A–H) Colonic sections stained with hematoxylin and eosin (H&E) (A–D) or alcian blue with H&E counterstaining (E–H) from mice treated with the indicated diet and/or antibiotics. Asterisks indicate goblet cells, which stain intensely with alcian blue but not H&E. (I, J) Boxplots of the average gland length for each treatment group (I) or the average number of goblet cells per gland (J). Datapoints plotted as circles had normal gland morphology, while those plotted as diamonds had hyperplasia. The mean value is plotted with a plus sign. *p*‐values are shown from a two‐way ANOVA analysis of diet*drug. Different letters indicate statistically significant differences between groups at *p* < .05 by post hoc analysis of ANOVA. Sample size is *n* = 6 (HG, LG), *n* = 7 (HGAbx), *n* = 9 (LGAbx).

The metabolic signatures associated with antibiotic‐induced dysbiosis in HGAbx mice suggested gastrointestinal inflammation as a potential mechanism underlying the acute mortality we observed. During necropsy of moribund HGAbx mice, colonic tissue was preserved and evaluated alongside colonic tissue from control HG and LG mice as well as LGAbx mice. These colonic sections were stained with hematoxylin and eosin (H&E) or alcian blue and were scored blindly by a pathologist (Figure 4A–H). Colonic gland lengths were measured (Figures 4I and S6A) and goblet cell numbers quantified (Figure 4J).

We observed that control HG and LG glands (also referred to as colonic crypts) had normal morphology and distribution of goblet cells (stained instensely with alcian blue), with LG glands being approximately 33% longer than HG glands and having approximately 64% more goblet cells (Figure 4A,B,E,F,I,J). HGAbx glands, in contrast, were hyperplastic and had a significant reduction in goblet cell numbers, relative to HG or LG glands (Figure 4C,G,I,J). There were two notable exceptions that were not hyperplastic and had similar gland lengths to control HG glands (Figure 4I, circles). These two mice did not die of cecal hemorrhage and lived past 90 weeks of age (see Figure 1B). We also observed that hyperlastic colons had much higher intraindividual variation in gland length than nonhyperplastic colons (Figure S6A).

Analysis of LGAbx colons revealed a complementary finding to the HGAbx colons. The majority of samples had normal morphology and numbers of goblet cells, with lengths and globlet cell numbers similar to HG control glands (Figure 4I,J, circles). Two samples were hyperplastic and had goblet cell loss (Figure 4I,J, diamonds). Both of these mice died with cecal hemorrhage, similar to those observed in HGAbx mice and were the only non‐HGAbx mice to die of apparent gastrointestinal disease. Therefore there was perfect correspondence between colonic hyperplasia and cecal hemorrhage‐associated mortality.

Dysbiosis is associated with colonic inflammation and decreased gut barrier function. We therefore assessed colonic inflammation histopathologically and via gene expression. Two of the HGAbx samples showed evidence of inflammation. One sample with hyperplasia had neutrophil infiltration in the submucosa, while another HGAbx sample without hyperplasia had a band of small lymphocytes between the gland and muscularis mucosa (Figure S6B,C). Otherwise, no obvious pathology was noted.

In order to further assess colonic inflammation, we collected colonocytes from scrapings at necropsy. At this time (48 weeks of treatment), only one HGAbx mouse had survived and thus was excluded from analysis. Of the inflammation‐associated genes that we analyzed in the colonocytes (TNF‐α, IL‐1β, and IL‐10), no significant differences were observed between groups (Figure S7A). We also evaluated a panel of gut barrier function genes (Ocld, Tjp1/ZO‐1, Tjp2/ZO‐2, Cldn1, Cldn2). Two genes were significantly increased between LG and LGAbx samples, Ocld and Cldn2, genes associated with increased and decreased gut barrier function, respectively (Figure S7B). We also assessed systemic inflammation in these same groups of mice and did not observe differences in plasma cytokine levels for IL‐10, IL‐6, TNF‐α, MCP‐1, or IL‐1β (Figure S8). Overall, our analyses did not reveal meaningful changes in inflammation and gut barrier function in LGAbx mice, thus indicating functional rescue from antibiotic‐induced dysbiosis.

## 4 DISCUSSION

Commensal bacteria in the gut promote host health through multiple mechanisms including modulating local and systemic immune function, microbial metabolite production, and prevention of pathogenic bacteria colonization. Ablation of commensal gut bacteria through oral broad‐spectrum antibiotic treatment has been shown to contribute to enteric infection,^41^ and long‐term or frequent use of oral antibiotics is associated with diseases ranging from obesity to depression.^7^, ^42^ Recent evidence suggests that diet may modulate the effect of antibiotics on microbial composition and function,^15^, ^16^, ^17^, ^18^, ^43^, ^44^ but the effect of this modulation on host health has not been explored in an aging context.

To determine the effect of the antibiotic‐diet interaction on host health, we fed aged mice an HG or LG diet with or without antibiotics. Unexpectedly we observed a previously unreported lethal antibiotic‐diet interaction in mice fed HG diets containing antibiotics (HGAbx), with a median survival of 4 months on treatment, compared to at least 12 months in mice fed a LG diet with antibiotics. A previous study by Hyoju et al. showed that mice fed a high‐fat diet (60% lard) and treated with cefoxitin and clindamycin died following a 30% surgical hepatectomy, normally a recoverable injury.^45^ Death was associated with damage to multiple organs, particularly the lungs, with evidence of septicemia caused by multiple‐drug‐resistant proteobacteria. Both the high‐fat diet and our HG diet led to changes in the microbiome and depletion of Bacteroidetes, along with weight gain and insulin resistance. Therefore, aging in our model may replace the need for the additional stress of surgery.

Upon gross necropsy, HGAbx mice had an enlarged and massively hemorrhagic cecum. No other signs of disease were observed. These observations suggest blood loss, sepsis, or both, as the primary cause(s) of death and implicate deteriorating gut health. Cecal hemorrhage in mice has only been previously described in response to cecal torsion,^46^ which was not observed in our mice, or through intentional direct intervention such as surgical perforation or genotoxicity studies.^47^, ^48^

We considered the possibility of colonic inflammation as a potential contributing factor for disease in HGAbx mice. To our surprise, we did not uncover evidence for massive inflammation in HGAbx mice, but we did observe colonic gland hyperplasia and loss of goblet cells, which correlated perfectly with lethality and cecal hemorrhage in HGAbx mice and two LGAbx mice. The hyperplasia we observed was reminiscent of the transmissible murine colonic hyperplasia that is caused by *Citrobacter rodentium* or pathogenic strains of *Escherichia coli*.^49^, ^50^ Gland hyperplasia is the result of repair of an earlier breach of the epithelial barrier and requires a TLR4‐MyD88‐dependent pathway.^51^ Neutrophils are thought to be important protective factors in preventing an overt bacteremic incident.^51^ We only observed neutrophil infiltration in one sample, suggesting that defective neutrophil function in aged mice could contribute to the high lethality that we observed.^52^ It is possible that inflammation preceded colonic hyperplasia and acute death. Future studies will be required to determine the early immune responses to antibiotic‐induced dysbiosis in the gut.

Redox potential of the gut is another factor to consider in the role of HG diet in antibiotic‐induced dysbiosis. Antibiotic treatment is known to cause disruption of redox dynamics, leading to increased redox potential.^53^ The increased redox potential was associated with high levels of aerobic bacteria such as *Enterobacteriaceae* and was increased following a high glucose diet, but was normalized following a high prebiotic diet.^18^, ^53^ The predicted metabolic functional profile of HGAbx mice is consistent with an aerobic, high redox potential gut, particularly the increased capacity for fatty acid biosynthesis and TCA cycle.

A key finding from our study was the prevention of antibiotic‐induced compositional and functional dysbiosis by an LG diet. Whereas antibiotic treatment in HG‐fed mice eliminated most commensal bacteria, as has been described previously,^21^ the LG‐fed mice maintained a large relative abundance of the genus *Bacteroides*, which consists of carbohydrate‐fermenting, anaerobic bacteria with functions known to promote health.^54^ It is possible that this population of bacteria supported gut epithelium integrity, reduced inflammation, and prevented severe infection of pathogenic bacteria. This interpretation is supported by our observation of increased numbers of goblet cells in LG‐fed mice compared to HG‐fed mice and only partial diminution of goblet cell numbers in LGAbx‐fed mice. Our taxonomic data indicated that 9/10 LGAbx and 0/10 HGAbx samples had high levels of a single amplicon sequence variant (ASV) that was most likely *Bacteroides thetaiotaomicron*, based on shotgun metagenomic sequencing results from mice in the study that were taken at a later age. However, exact species identification of *Bacteroides* within the samples in our study was not possible via our 16S sequencing methodology.

There is evidence that carbohydrate composition can modify the effects of various antibiotics on bacteria composition. Inulin supplementation modified the effect of ampicillin and gentamycin on total bacteria abundance and abundance of *Bacteroides* and *Bifidobacterium* in anaerobic batch cultures of fecal bacteria.^16^ In contrast, a diet deficient in microbial‐accessible carbohydrates prevented the recovery of *Bacteroidaceae* in mice following ciprofloxacin treatment.^17^ A fiber‐free diet prevented the recovery of Bacteroidetes and Firmicutes in human volunteers treated with vancomycin and neomycin, whereas those consuming a standard omnivorous diet had complete recovery of these phyla at 7 days posttreatment.^55^ In concurrence with our findings, *Bacteroides thetaiotaomicron* has decreased susceptibility to amoxicillin when grown on media containing polysaccharides, and glucose supplementation during amoxicillin treatment reduced the abundance of this taxon in mice.^43^

Another consideration is the effect of diet on host metabolism to modify antibiotic treatment outcomes. A study in rats found that a high‐fat diet modified the effect of vancomycin and ciprofloxacin/metronidazole on glucose homeostasis and liver function causing long‐term defects, even after recovery, compared to rats fed control chow diets.^56^ We showed here, and in previous work, that the HG diet leads to impaired glucose tolerance and increases postprandial hyperglycemia, which is associated with encroachment of gut microbiota into the inner mucus layers of the colon and contributes to gut permeability.^57^, ^58^ Similarly, streptozotocin‐induced hyperglycemia increased the severity of antibiotic‐induced changes in gut microbiome composition and function as well as susceptibility to enteric infection.^59^

The gut microbiome contributes to the maintenance of gut epithelial integrity through multiple mechanisms, including immune function and tight junction expression regulation, regulation of mucin layer thickness, production of short‐chain fatty acids and other metabolites, and competition with pro‐inflammatory microbes.^60^ We were unable to measure these effects directly due to the unexpected and rapid loss of animals in the HGAbx group. However, we did probe predicted microbial functional capacity for these functions, specifically focusing on tryptophan metabolic pathways, secondary bile acid pathways, and short‐chain fatty acid pathways. This group of metabolites has important impacts on glucose metabolism, lipid metabolism, inflammation, and gut‐organ axis signaling.

Dysbiosis in HGAbx mice was associated with the loss of bile salt hydrolase, which is necessary for the production of secondary bile acids. Secondary bile acids are generally associated with anti‐inflammatory roles, particularly in the context of inflammatory bowel disease, where levels of secondary bile acids are reduced and conjugated bile acids are high.^61^ Anti‐inflammatory effects of secondary bile acids are mediated in part through activation of the TGR5 receptor in immune cells, along with maintenance of mucosal integrity and suppression of Th1 activation.^62^ Another consequence of high levels of taurine‐conjugated bile acids and low levels of secondary bile acids in HGAbx mice would be antagonism of the FXR receptor, which may explain some of the reduction in adiposity compared to HG mice.^63^ Analysis of tryptophan metabolism potential in HGAbx mice compared to control mice and LGAbx mice revealed an increase in kynurenine degradative pathways and a decrease in indole degradative pathways. Increased kynurenine is associated with aging, muscle and bone loss, and inflammation in humans.^64^, ^65^, ^66^ Conversely, inhibition of kynurenine pathways leads to extended lifespan and healthspan in model organisms.^67^ Nevertheless, kynurenine metabolism leads to the production of a number of important metabolites such as kynurenic acid, which is regarded as neuroprotective, and metabolic precursors of NAD+, an important energy source.^68^ Our data predict that kynurenine metabolism in the HGAbx mice would be shunted toward the production of quinolinic acid, a neurotoxin, from anthranilate. Similarly, our data suggest that indole pathway generation via tryptophanase is reduced in HGAbx mice. Indoles and their downstream metabolites have been linked to improved gut barrier function and anti‐inflammatory functions.^69^ However, indole can also generate idoxyl sulfate, a uremic toxin, and oxindole, which can negatively impact brain function. Thus, the net concentration of all kynurenine and indole metabolites needs to be considered to understand the net impact of altered microbiota metabolism. While we did not observe differences in short‐chain fatty acid production by diet or antibiotic treatment, we did note increased propionate degradation capacity in the HGAbx group, which could potentially deplete the available pool of propionate. Our results need to be corroborated with metabolomic data, but they do point toward altered metabolite signaling as a possible functional change leading to increased mortality in HGAbx mice.

Metagenomic analysis of LGAbx mice suggested that most metabolic alterations associated with the HGAbx treatment were not observed in LGAbx mice. However, this data, on its own, does not prove that LG diet prevented antibiotic‐induced dysbiosis. Therefore, we relied on measures from colonic tissue and plasma to evaluate physiologically relevant hallmarks of dysbiosis, intestinal permeability, and inflammation. Evaluation of colonic scrapings in LGAbx mice indicated that tight junction proteins were not different in LGAbx mice compared to control HG and LG mice. We did observe an increase in occludin and claudin‐2 in LGAbx mice. Occludin stabilizes the tight junction protein complex. Increased expression may lead to increased tight junction stability and decreased permeability.^70^ Additionally, the expression of occludin is negatively regulated by tumor necrosis factor‐α (TNF‐α), which is released in response to microbial‐associated peptides. The observed increase in occludin and lack of change in TNF‐α suggests that the LGAbx group did not experience increased microbial translocation. Claudin‐2 is a tight junction protein that forms a cation‐selective channel, allowing paracellular permeability to water and cations. The expression of claudin‐2 is promoted in many disease states and has been implicated in enteric infections.^71^ However, we did not observe any effect of the LGAbx treatment on colonic or plasma cytokines associated with tissue and systemic inflammation.

Our study has strengths and limitations. Strengths of the study include the use of well‐validated micronutrient and macronutrient‐matched diets that differ only in starch composition. In this respect, we can be confident that differences in microbiota and gut health are not related to other dietary differences. There are several limitations in our study. First, we only studied male mice, thus the generalizability of our findings to female mice is uncertain. Second, our microbiome analysis relied on 16S rDNA sequencing and inference of function from compositional data. Thus, we do not have precise species‐level information in many cases and have not directly measured genes associated with bacterial metabolic pathways, although the algorithm has been shown to produce nearly the same interpretations as metagenomic sequencing.^32^ Third, our statistical methodology relied on normalization of data, which has the potential to introduce bias, especially in antibiotic‐treated samples, which are zero‐inflated.^72^ We attempted to mitigate this bias using alternative differential abundance methodologies, as recommended by Nearing et al. and robust filtering criteria to reduce the false positive rate.^73^ Finally, we were limited in what samples we were able to retrieve from HGAbx‐treated mice, as their deaths came suddenly. Future studies are required to address these shortcomings and provide clear mechanisms underlying lethality in HGAbx‐treated mice.

Overall, we found that the interaction of the HG diet and oral antibiotics resulted in a significant decrease in survival, gut dysbiosis, and colonic hyperplasia. These phenotypes were dramatically reduced by feeding an LG diet containing high‐amylose corn starch. Our study is well‐aligned with other recent findings that prebiotic‐rich diets are capable of ameliorating antibiotic‐induced dysbiosis and should prompt rethinking of conventional medical advice surrounding antibiotic use, especially in older adults.

## AUTHOR CONTRIBUTIONS

Kelsey M. Smith and Sheldon Rowan designed approach, generated primary manuscript, analyzed data, and generated figures. Kelsey M. Smith, Sarah G. Francisco, Ying Zhu, Meredith L. Davis, and Donald E. Smith performed in vivo experiments. Meredith L. Davis and Jimmy W. Crott performed and analyzed colonic gene expression data. Tanya LeRoith and Laxmi Yeruva evaluated and analyzed colonic histology. Kelsey M. Smith and Kathryn Barger analyzed microbiome data and biostatistical analyses. Kelsey M. Smith, Jimmy W. Crott, Allen Taylor, Andrew S. Greenberg, Laxmi Yeruva, and Sheldon Rowan revised the manuscript. Sheldon Rowan secured funding for the project with additional funding provided by Allen Taylor, Andrew S. Greenberg, and Laxmi Yeruva.

## DISCLOSURES

The authors report there are no competing interests to declare.

## Supporting information


Data S1.

## Data Availability

The data that support the findings of this study are available from the corresponding author, S.R., upon reasonable request. Full microbiome sequence data are available from the MG‐RAST metagenomics analysis server: https://www.mg‐rast.org/linkin.cgi?project=mgp97315.
