# Supplementary material for: Dietary prevention of antibiotic‐induced dysbiosis and mortality upon aging in mice
Source: FASEB J. 2024 Dec 10;38(23):e70241. doi: 10.1096/fj.202402262R (PMC11629448; doi:10.1096/fj.202402262R)
Supplement: Supplementary file 1 — Data S1. [file FSB2-38-e70241-s001.pdf]

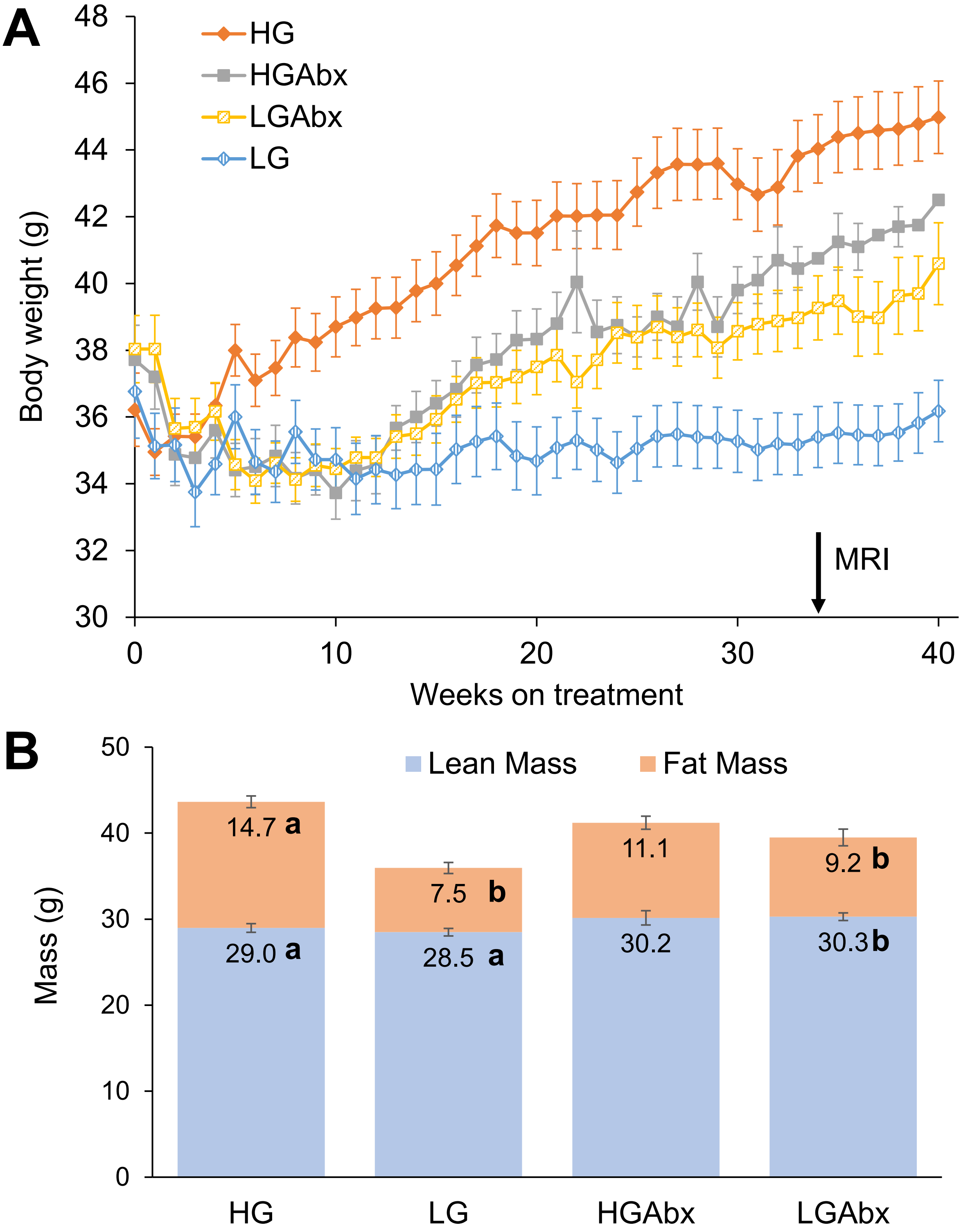

Figure S1. Body weight and composition. (**A**) Body weight during aging in the treatment groups. Error bars indicate SEM. Arrow indicates 36-week data point when MRIs were performed (**B**) MRI data of body composition showing lean body mass and fat mass. Error bars indicate SEM. Different letters indicate statistically significant differences of $p < 0.05$ from Tukey's HSD post-hoc analysis of ANOVA 1-way comparison. HGAbx mice were excluded from statistical analysis due to small sample size. Sample size: HG=15, LG=14, HGAbx=2, LGAbx=13.

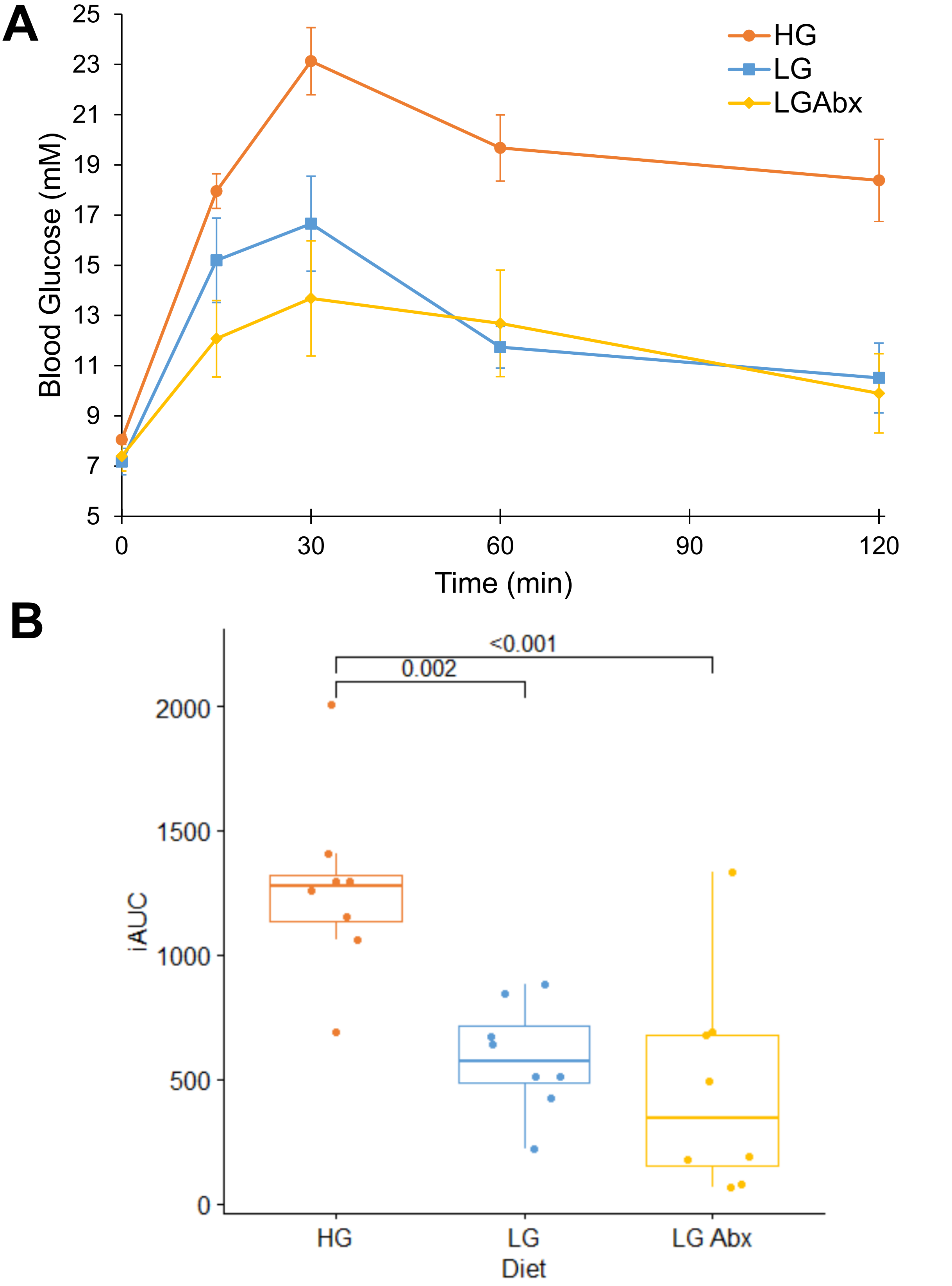

Figure S2. Glucose tolerance test results. Intraperitoneal glucose tolerance tests (IPGTT) were performed on mice treated with HG, LG, or LGAbx diets at 37-weeks of treatment. (A) Results of the IPGTT show glucose intolerance in HG-treated mice but not LG or LGAbx-treated mice. (B) Statistical analysis of GTT data using incremental area-under-the-curve of the GTT plot (AUC) shows significantly increased AUC in the HG group relative to LG or LGAbx group. Statistics shown are p-values from Tukey's HSD post-hoc analysis of ANOVA 1-way comparison. Sample size is n=8.

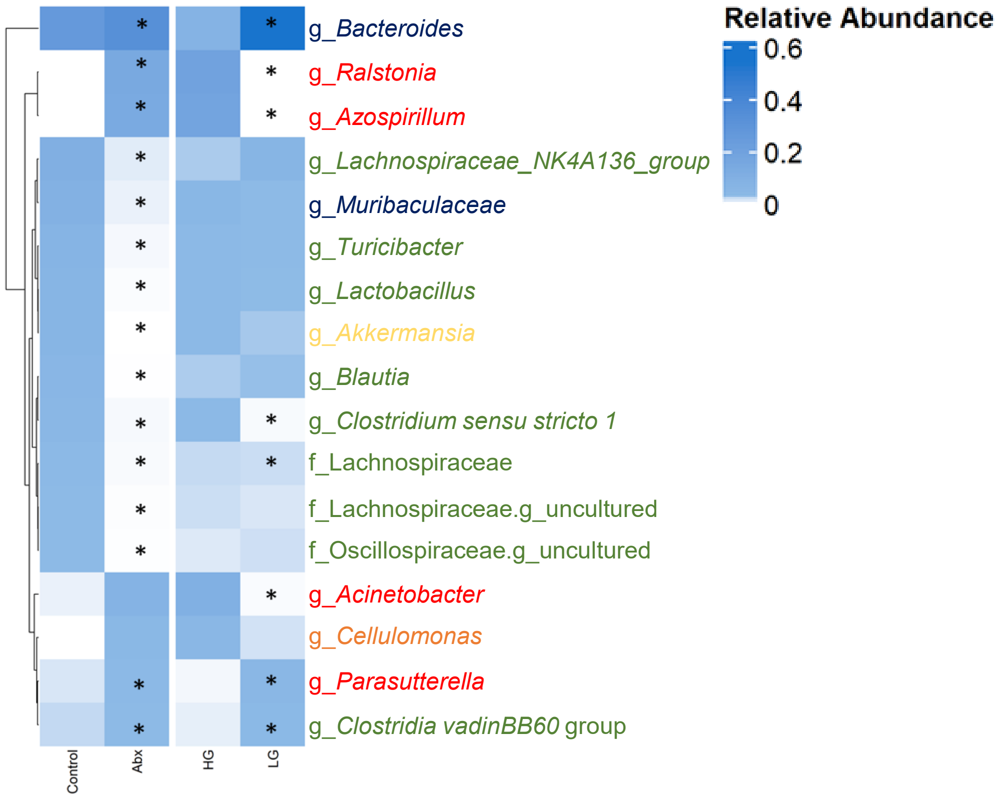

Figure S3. Heatmap of average relative abundance of significantly differentially abundant taxa by main effect. Taxa are filtered to those with an average relative abundance of at least 1% in at least one comparison group. Asterisk indicates significant main effect comparison (adjusted p-value < 0.25). Sample size n=10 Control; n=20 Abx; n=15 pooled diet group.

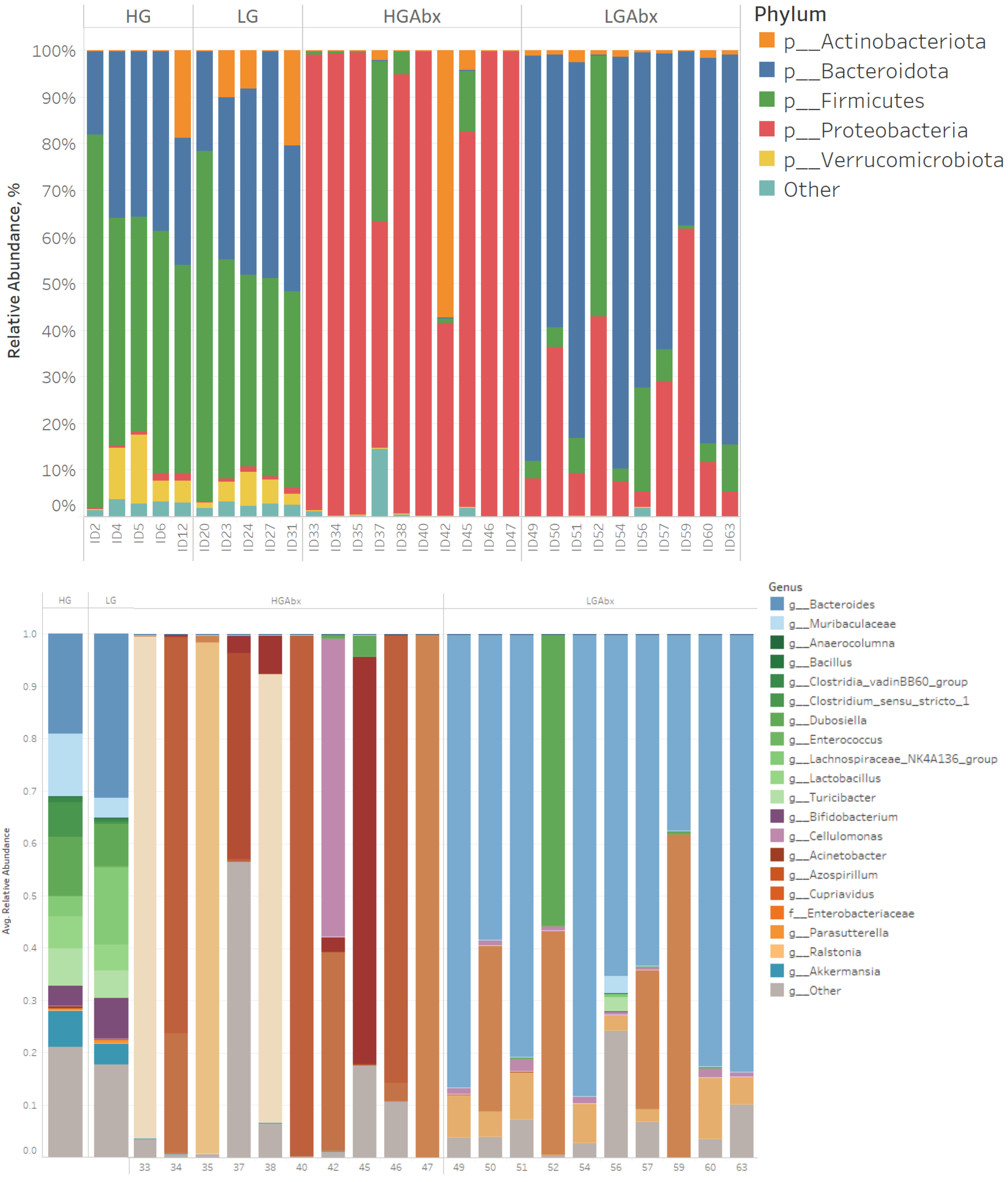

Figure S4. Microbiota composition for individual samples at the phylum level (top) or genus level (bottom).

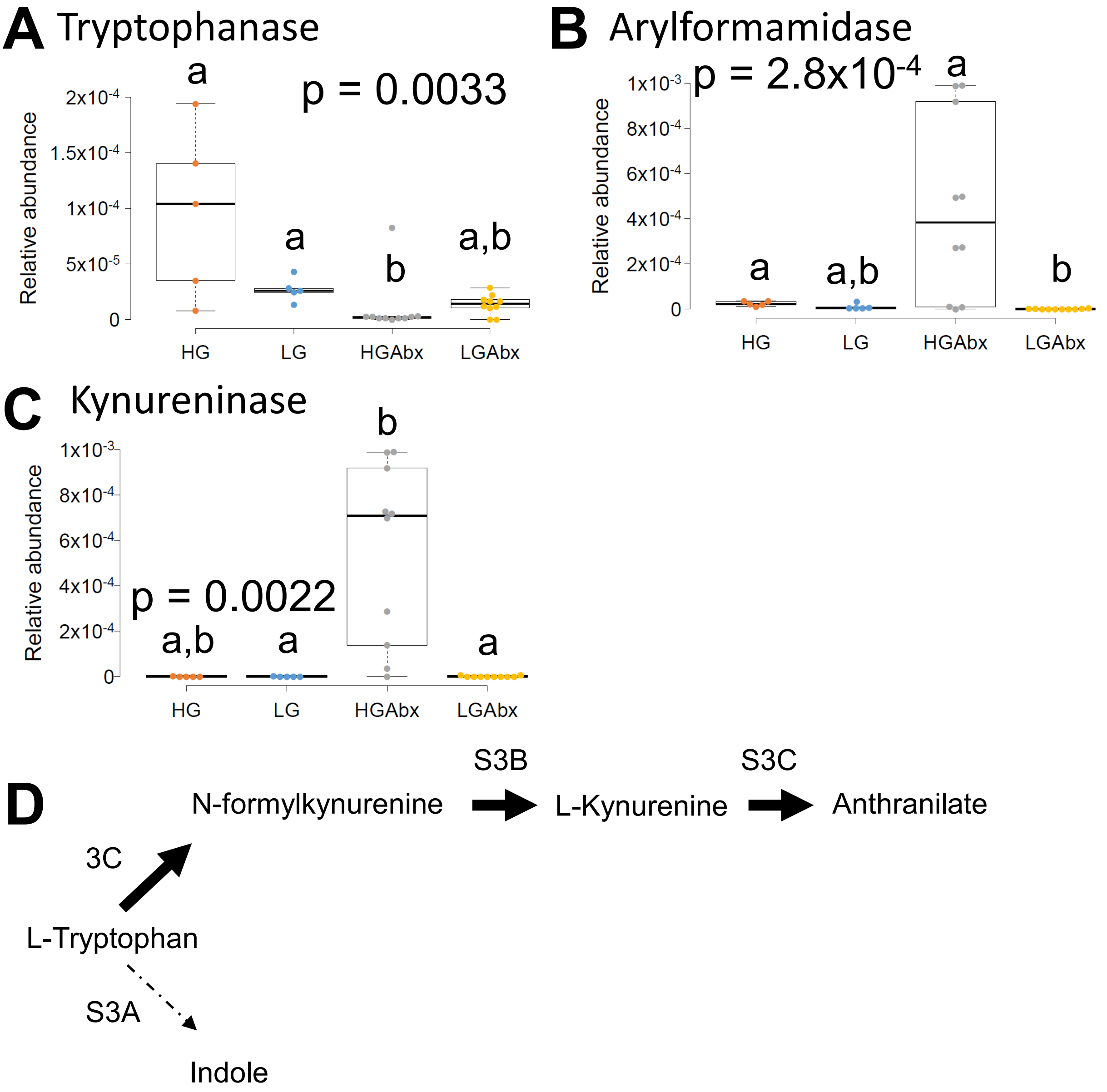

Figure S5. Differentially abundant genes involved in tryptophan degradation pathways. (**A-C**) Boxplots of genes encoding tryptophanase (**A**), arylformamidase (**B**), or kynureninase (**C**). P-values are indicated for Kruskal-Wallis analysis with different letters indicating $p < 0.05$ from post-hoc analysis using Dunn's test with Holm's correction. Sample size n=5, LG, HG; n=10 LGAbx, HGAbx. (**D**) Schematic showing enrichment in HGAbx group for genes directing degradation of tryptophan toward kynurenine pathway compared to indole pathway. Enzymes are as indicated in Fig. 3 or Fig. S5.

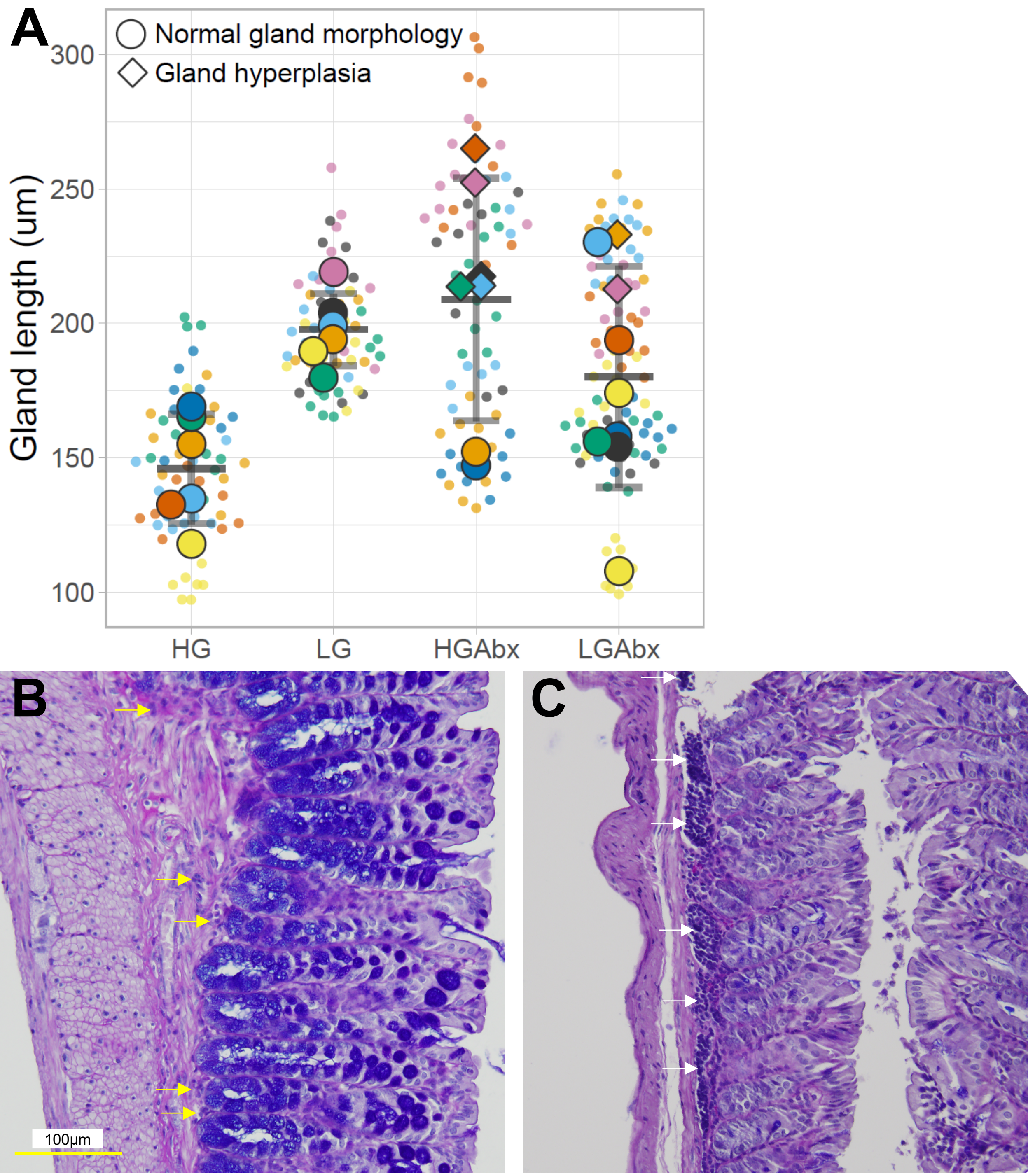

Figure S6. Colonic hyperplasia and inflammation. (**A**) Superplot of colon gland lengths, showing the range of individual data points. Hyperplastic glands showed greater overall variability within measurements. (**B-C**). Alcian blue photographs of HGAbx samples that showed evidence of inflammation. (**B**) Neutrophil infiltration (yellow arrows) in the submucosa of a sample with hyperplasia that was 99.98% *Enterobacteriaceae*. (**C**) Bands of small lymphocytes (white arrows) in a sample without hyperplasia that was 96.3% *Ralstonia*.

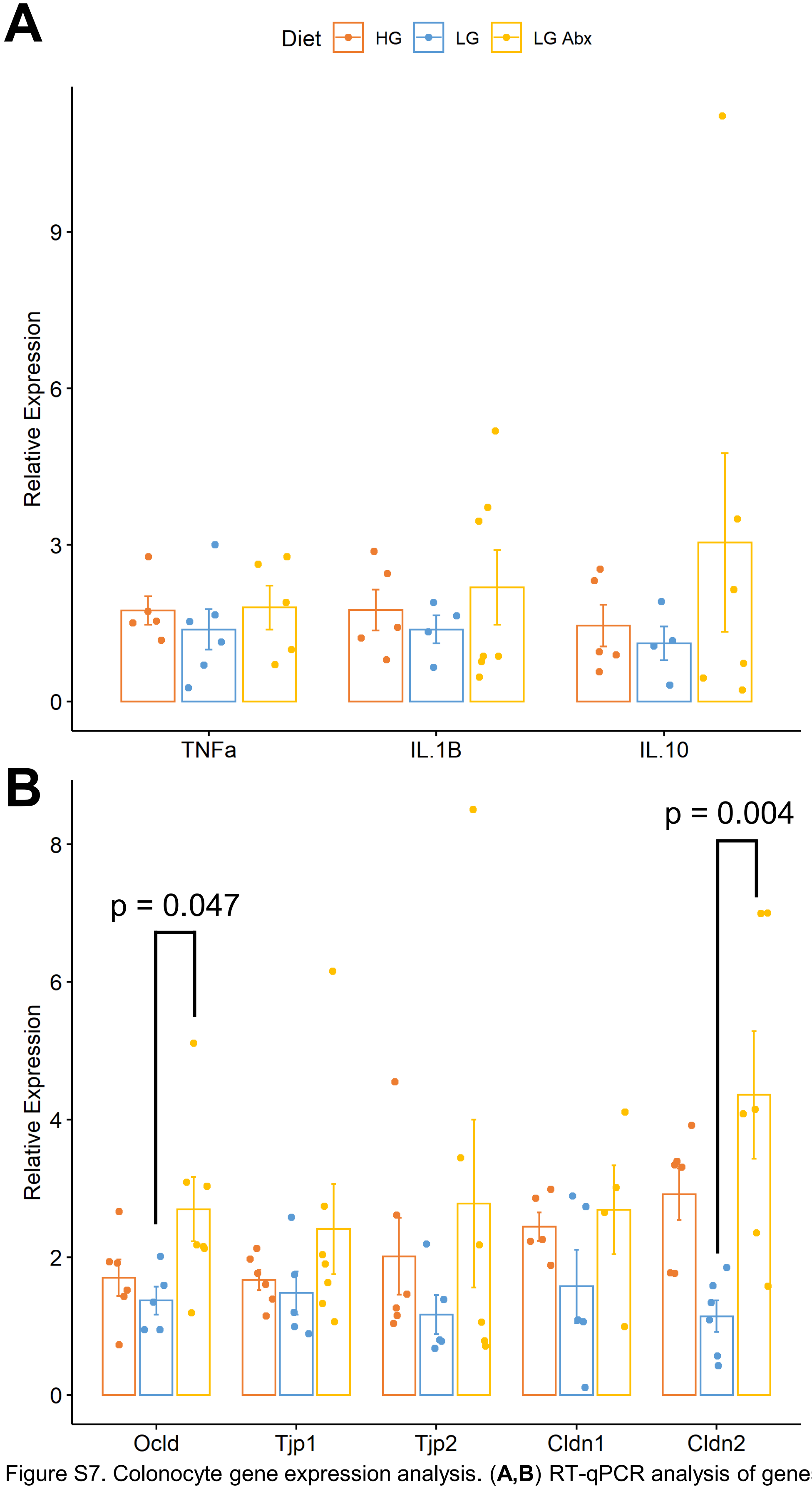

Figure S7. Colonocyte gene expression analysis. (**A,B**) RT-qPCR analysis of genes associated with inflammation (**A**) or permeability (**B**) in colonocytes from the indicated groups, normalized versus Gapdh. p-values are indicated for significantly different pairs, determined via Krusal-Wallis followed by Dunn's pairwise comparisons with Holm's correct. Bars indicate mean +/- SEM. Sample size is n=6 per group.

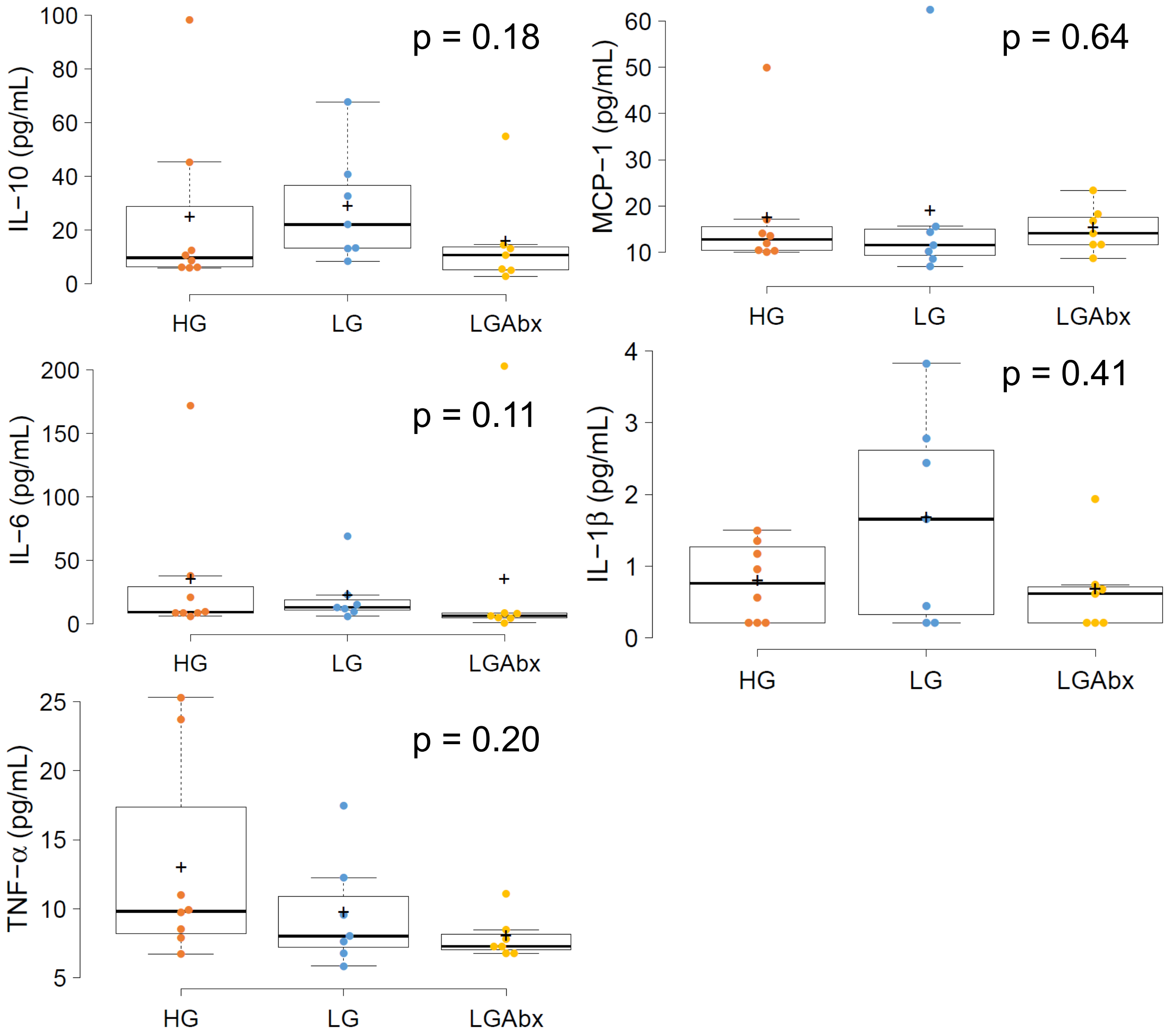

Figure S8. Analysis of plasma cytokine levels in fasting plasma from mice fed HG, LG, or LGAbx diets, as indicated. p-values are determined by Kruskal-Wallis test. Sample size is n=8, HG; n=7 LG, LGAbx.
